# The sequence variation of mitochondrial tRNA tyrosine and cysteine among Iranian women with idiopathic recurrent miscarriage: A case-control study

**DOI:** 10.18502/ijrm.v21i7.13894

**Published:** 2023-08-23

**Authors:** Elham Mojodi, Alimohammad Mosadegh Mehrjardi, Yasaman Naeimzadeh, Nasrin Ghasemi, Ali Falahati, Seyed Mohammad Moshtaghioun

**Affiliations:** ^1^Department of Biology, Faculty of Science, Yazd University, Yazd, Iran.; ^2^Department of Traditional Pharmacy, Faculty of Traditional Medicine, Tehran University of Medical Sciences, Tehran, Iran.; ^3^Department of Molecular Medicine, School of Advanced Medical Sciences and Technologies, Shiraz University of Medical Sciences, Shiraz, Iran.; ^4^Abortion Research Center, Yazd Reproductive Sciences Institute, Shahid Sadoughi University of Medical Sciences, Yazd, Iran.

**Keywords:** Recurrent early pregnancy loss, mtDNA, SNPs, Heteroplasmy.

## Abstract

**Background:**

Recurrent miscarriage is one of the most prevalent reproductive diseases. This phenomenon has several reasons, including maternal, hormonal, immunological, and parental genetic factors. Idiopathic recurrent miscarriage (IRM), with no distinctive etiology, involves about half of the recurrent miscarriage cases. Some mutations in mitochondrial DNA can lead to miscarriage. Mitochondrial tRNA (mt-tRNA) mutations cause nearly half of the mitochondrial disorders.

**Objective:**

To identify *mt-* *tRNA ${}^{Cys\&Tyr}$* gene mutations in Iranian women with IRM.

**Materials and Methods:**

In this case-control study, 100 Iranian women with IRM and 100 women as control without any history of miscarriage were investigated by polymerase chain reaction-single strand conformation polymorphism technique followed by gene sequencing. Bioinformatics analysis were done using human mitochondrial genome database, molecular evolutionary genetics analysis, mammalian mitochondrial-tRNA, etc.

**Results:**

Results showed 4 mt-tRNA mutations including 1 cysteine mt-tRNA mutation (5824C > T) and 3 tyrosine mt-tRNA mutations (5868T > A, 5849C > T, and 5836T > C) in our cases.

**Conclusion:**

Amongst the 4 mutations found, one was novel that is still not reported. Our bioinformatics analysis revealed that these mutations can be pathogenic. They occurred in tRNA-conserved regions and their secondary structure was changed, which can result in mitochondrial dysfunction. Mutations of these genes may help in the assessment of IRM. Further study of all 22 mt-tRNAs possible mutations is recommended to describe their etiologic role in IRM.

## 1. Introduction

A miscarriage is a gestation that ends spontaneously before the fetus is old enough (1). It occurs before the 20 ${}^{\text{th}}$ wk of pregnancy (2). Sporadic miscarriage (i.e., 1-2 miscarriages) is considered a primary defect of the survival progression in the abnormal embryo. Although “recurrent pregnancy loss”, as a traditional definition, is 3 or more sequential miscarriages; however, the American Society of Reproductive Medicine has recently defined it as 2 or more miscarriages. So “recurrent miscarriage” (RM) has replaced the traditional term as a precise definition (3). Nearly 15-20% of clinically distinctive gestations end in miscarriage, and 5% of couples are affected by RM. There are several possible reasons for RM to occur, such as parental genetic factors, maternal hormonal, anatomic, immunologic, thrombophilic, microbiological factors, sperm quality, and lifestyle. More than 50% of cases are without etiology and they are assortment as idiopathic RM (IRM) or unexplained recurrent spontaneous abortion (4, 5).

In couples with RM, maternal causes are dominant (5). Increasing maternal age, which causes defects such as chromosomal abnormalities and embryo aneuploidies are observed in RM more than in sporadic miscarriage (6, 7). In addition to maternal roles, paternal factors such as abnormal sperm play an important role in RM. Semen with high DNA fragmentation, caused by oxidative stress, is one of the known causes of miscarriage and poor embryonic development (8).

Some studies revealed a higher frequency of mitochondrial DNA (mtDNA) variations in women with RPL (9-11). Mitochondria are intracellular multifunctional organelles with their own genome, which contains a high density of genes and lacks introns, named mtDNA (12). They are responsible for metabolism, reaction to oxidative stress, and energy production (i.e. adenosine triphosphate [ATP]) by oxidative phosphorylation system and electron-transport chain (13). 2 important mitochondrial performances including oxidative phosphorylation and apoptosis are considered as the RM etiology. Damaged oxidative phosphorylation can lead to early pregnancy loss. Also apoptosis is a dynamic and interactive biological procedure which occurs in all stages of embryogenesis (14).

As mentioned above, in the human mitochondrion, 22 tRNAs are encoded by mtDNA. The tRNAs contain about 10% of total RNA and are essential components for the protein synthesis (15). mt-tRNA mutations account for 50% of the reported mtDNA mutations, due to this reason *mt-tRNA* genes are considered hot spots for mitochondrial disorders (15, 16). Gene variations have the main role in increasing the risk of miscarriage in patients with IRM (17).

In this study, we have investigated mt-DNA variations in *Cys* and *Tyr* *mt-tRNA* genes among Iranian women with IRM.

## 2. Materials and Methods

### Population

Participants included 100 women who had referred to the Yazd Reproductive Sciences Institute, Yazd, Iran with a history of RM, and those who had experienced at least 3 miscarriages with IRM. Several criteria were considered for selecting RM cases such as participants should have 3 or more consecutive RMs, 6 months must have passed since their last miscarriage, and the etiology of miscarriages must be unknown. The cases with intentional abortions, physical disorders, chromosomal rearrangements, urinary or genital tract infections, and a history of taking certain medications (e.g., contraceptive drugs) were omitted from the study. Also, 100 women as control cases without any history of RM who had 2-3 live birth were chosen and compared with RM cases to identify single nucleotide variations in *mt-* *tRNA ${}^{Cys\&Tyr}$* genes. About 5 ml of blood was taken from each participant and poured into tubes containing ethylene diamine tetra acetic acid tubes and stored at -20 C.

### Primer designing

The primers were designed using the gene sequences recorded in the GenBank database by Gene Runner software version 3.05 (Hastings Software Inc. Hastings, NY, USA, http://www.generunner.com) and BLAST tool (Table I). *Mt-tRNA ${}^{Cys\&Tyr}$* genes are side by side in human mitochondrial genome and amplify in one PCR product by the primers as a 281 bp product.

### DNA extraction

DNA extraction was performed by QIAGEN kit (BIORAIN Co., Tehran, Iran) from blood samples. The quality and quantity of extracted DNA were measured by agarose gel electrophoresis and Nanodrop apparatus. Finally, the polymerase chain reaction (PCR) was performed.

### PCR

In this research, amplification of our target gene fragment (amplicon) was done using PCR technique. Our product length was 281 bp. Several factors are needed to amplify the amplicon, such as primers, DNA template, Mastermix, and water. Mastermix kit contains dNTPs, MgCl2, PCR buffer, and taq-DNA polymerase enzyme. All PCR reactions were accomplished in a total volume of 25 μl with 3 µL template DNA, 2 µl forward and reverse primers, 12.5 µl 2X Mastermix, and 7.5 µl water. A PCR program for amplifying the target fragments was setup as follows, initial activation at 95 C for 5 min, followed by 35 thermal cycles at 95 C for 30 sec, 65 C for 40 sec, and 72 C for 30 sec, and finished with 5 min, final extension, at 72 C.

### Single-stranded conformation polymorphism technique

This method is based on the 3-dimensional and spatial structure of different single-strand DNA sequences. Double-stranded DNA is denatured by heat and then cooled quickly to obtain single-stranded DNA. We used polyacrylamide gel electrophoresis with a high-resolution detection for recognizing the probable base variations in our PCR amplified DNA fragments.

### PCR for mutant samples sequencing

After performing single-stranded conformation polymorphism, 25 samples with different shifted band patterns were sent to Pishgam Biotech Company, Tehran, Iran for sequencing. PCR was done again for samples that are suspected to mutation. Amplified product quality was evaluated on a 1.8% agarose gel electrophoresis. Samples with a single band without any unspecific product were acceptable.

### Bioinformatics and sequencing results analysis 

We used various databases for genes sequences and positions and sequence alignment, such as the GenBank database (to obtain the FASTA format of DNA sequences), BLAST online software (to compare DNA nucleotide sequences), MITOMAP mt-DNA database (based on a scoring system, the protection of the desired location, frequency of mutation, heteroplasmy, mutation or polymorphism are checked), MAMIT-tRNA database (investigation of structural features of mt-tRNA for recognizing point mutant-related human diseases in *tRNA* genes), MFOLD webserver (predicting possible structures of tRNAs), MEGA4 and CLC software (detecting mutations and comparison of different sequences), and Chromas software (displaying sequences chromatograms from sequencing, alignment).

**Table 1:** Specifications of primers


**Primer name **	**Primer sequencing**	**Primers length**	**Annealing temperature**	**Product length**
**Forward **	5 ' AGCACCCTAATCAACTGGCT3 '	58	
**Reverse**	5 ' CAGCTCATGCGCCGAATAAT3 '	20 nucleotide	58.2	281 bp

### Ethical considerations

All the women who participated were explained and encouraged for this study and their verbal consent was received. The study was approved by Research Ethics Committee of Yazd University, Yazd, Iran (Code: IR.YAZD.REC.1400.091).

## 3. Results

### Results of cysteine and tyrosine mt-tRNAs gene fragment sequencing

After receiving the gene fragments sequencing results from Pishgam Biotech Company and analyzing the results, 4 mutations were identified, including 1 cysteine and 3 tyrosine mt-tRNAs mutations.

According to the GenBank database review, the *MT-TC* gene is on mtDNA at positions 5761 to 5826 (66 bp). As mentioned, investigation of sequencing graphs and their alignment confirmed the existence of one mutation in this fragment that was a nucleotide replacement (5824C > T) in the CCA stem region of mt-tRNA that was found in case number 90.

**Figure 1:**
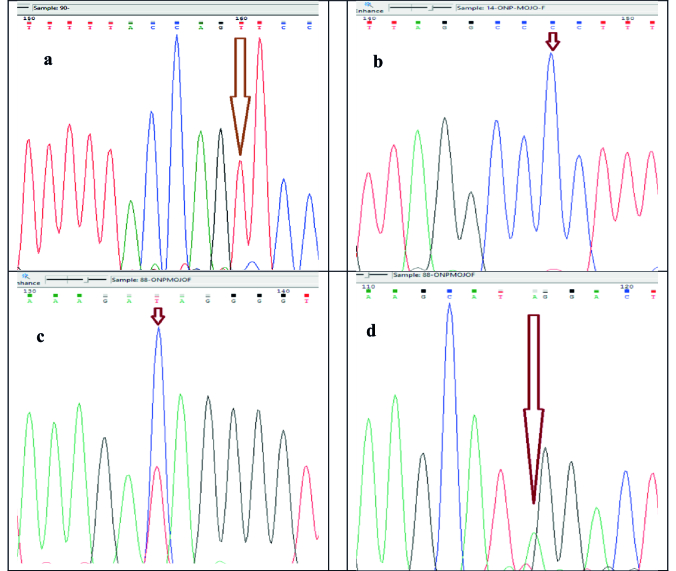
Chromatogram of resulting sequences: a) Cysteine mt-tRNA sequence related to C > T replacement in nucleotide 5824 of case number 90, b) Tyrosine mt-tRNA sequence related to T > C mutation in nucleotide 5836 of cases number 14 & 66, c) Tyrosine mt-tRNA sequence related to C > T mutation in nucleotide 5849 of case number 88, d) Tyrosine mt-tRNA sequence related to T > A homoplasmic mutation in nucleotide 5868 of case number 88.

*MT-TY* gene is located on mtDNA at positions 5826 to 5891 (66 bp). Like the abovementioned sequences, the investigation of sequencing graphs and their alignment, confirmed the existence of 3 mutations in this fragment. The first mutation was 5836T > C, which occurred in the CCA stem region of tyrosine mt-tRNA. This mutation was identified in cases 14 and 66. The second mutation was a heteroplasmic mutation that occurred in the CCA stem region of tyrosine mt-tRNA (5849C > T) and was found in case number 88. The third one was a homoplasmic mutation located in the T stem of tyrosine mt-tRNA (5868T > A). It was also found in case 88. The sequencing results for these 3 cases are presented in figure 1.

### Investigation of mutation position conservation by MAMIT-tRNA website 

To investigate the probable pathogenicity of detected mutations, we first check the conservation situation of the mutations points in their target region. If the region where the mutation occurred is the same in normal individuals and other mammals, then that area is considered highly conserved. Thus, mutations in this region can cause pathogenesis.

### Predicting the secondary structure of normal and mutant tRNAs using MFOLD webserver

**Figure 2:**
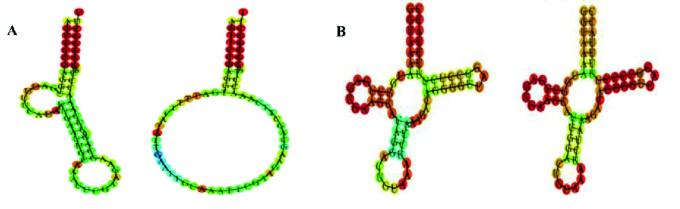
Results of secondary structure prediction of A) Left: Normal cysteine mt-tRNA (minimum free energy: -13/50), right: 5824C > T mutant cysteine mt-tRNA (minimum free energy: -12/80), B) Left: Normal tyrosine mt-tRNA (minimum free energy: -12/30), right: 5836T > C mutant tyrosine mt-tRNA (minimum free energy: -16/60) using MFOLD webserver.

This website predicts the secondary structure of normal and mutant tRNAs, based on the minimum free energy. DNA sequences of cysteine and tyrosine tRNAs were downloaded from the GenBank database. These sequences were converted to RNA using the sequence editor site. Then this sequence was transferred to the MFOLD webserver to predict possible structures with changes in the target tRNA. Replacements and deletions are applied, and their conformational differences with the original tRNA are checked (Figure 2).

## 4. Discussion

Mitochondrial dysfunctions can result from either mitochondrial or nuclear mutations. They may arise from impaired mitochondrial gene transcription and translation. Impaired translation can be caused by mutations in mt-tRNAs. Most the tRNA mutations are harmless compared to pathogenic mutations. However, mtDNA contains over 5-10% of the mutations, and point-mutations in the *mt-tRNA *genes are responsible for most mitochondrial disorders (18-20). Lack of energy in different organs leads to multiorgan dysfunction, which results in the variable appearances of mitochondrial disorders such as cardiac and skeletal myopathies, cognitive impairment, nephropathies, epilepsy, endocrinopathies, and hepatopathies (21). In this research, we found 4 mitochondrial tRNA mutations among Iranian women with IRM. One mutation in cysteine mt-tRNA and 3 mutations in tyrosine mt-tRNA. One of tyrosine mt-tRNA mutations was novel (5868T > A). Our bioinformatic analyses have revealed that the regions containing mutations were conserved in mt-tRNAs and so these mutations could be pathogenic. Because they can alter mt-tRNAs structures and functions, they probably cause mitochondrial dysfunction such as impaired apoptosis in placental tissues that can cause IRM.

tRNA has a cloverleaf secondary structure. It is folded into the inverted L-shape as a tertiary structure via interactions between the T and D arms (22). In cytoplasmic tRNAs, the D-arm and the T-arm contain conserved sequences, but these are not conserved in mt-tRNAs. The anticodon loop has 3 anticodon bases that match mRNA codon for protein translation. The acceptor stem that has a 5 ' -CCA-3 ' sequence added to the mature 3'-end is charged with the proper amino acid. The mt-tRNAs frequently have an `abnormal' secondary structure compared with their cytoplasmic counterparts (18, 23). Point mutations that occur in mt-tRNAs can impact their structure, folding, 3-dimensional stability, and aminoacylation. These mutations may either be pathogenic or polymorphic occuring in different parts of the mt-tRNAs, such as loops and stems. Polymorphic neutral mutations usually have no or low impact on the structure or function of mt-tRNA and can be found in all 22 mt-tRNAs. These changes can cause impaired cellular respiration or activity reduction of the respiratory chain complexes. Mutations can occur in different parts of mt-DNA, but the most functional destructive influences on mt-tRNAs are point mutations on the anticodon bases (15). As mentioned, 4 mutations were detected in this study in cysteine and tyrosine mt-tRNAs. The genes encoding these tyrosine and cysteine tRNAs are located on the light strand of mtDNA (24). One mutation was related to cysteine mt-tRNA, and 3 mutations were related to tyrosine mt-tRNA. One of these mutations was novel, and one of them was heteroplasmic. Cells with the same mtDNAs are named homoplasmic, and 2 mtDNA genotypes in identical cells are known as heteroplasmy. It is worth noting that most women with mitochondrial disease are heteroplasmic, and one of the mtDNA genotypes contains pathogenic mutation (10, 25). The mutation 5824C > T in cysteine mt-tRNA was observed only in one of the cases with IRM. This mutation occurred in the CCA stem region in the AGCTCCG sequence. This region is fully conserved and it is one of the first nucleotides involved in CCA stem hydrogen bonds. This sequence is very important for mt-tRNA function, and mutation in this region leads to biosynthesis impairment (26). Conservation means that if there has been a change (deletion, replacement, etc.) during evolution, they have been removed from the population. So, this sequence remains conserved, and if the nucleotide changes but the organism survives, it will be recorded as a mutation. This mutation changed the natural structure and main function of RNA, and as shown in figure 2, Δ G of the mutant mt-tRNA structure was changed to a more stable state compared to its natural structure. As a result, the steady state of RNA was altered, disrupting protein synthesis. These events, in turn, disrupt mitochondrial function. Therefore, the possibility of the pathogenesis of these mutations is suggested.

Cysteine, one of the sulfur-containing amino acids, has several important roles in cells. In general, the biological significance of sulfur-containing amino acids (cysteine, methionine, homocysteine, and taurine) plays a key role in cellular energy regulation. Cysteine plays a main role in several vital cellular processes, such as oxidative stress response, protein synthesis, structural and regulatory changes in proteins, and iron-sulfur (Fe-S) cluster biogenesis. Cysteine transport is essential for regulating cellular cysteine biosynthesis and adjusting the accessibility of sulfur for mitochondrial metabolism. Empirical evidence indicates that cysteine can infiltrate the lipid bilayer membrane of the cell and can penetrate mitochondria through special mechanisms of cellular cysteine uptake (21). The 3 mutations that occurred in tyrosine mt-tRNA were as follows: the first mutation, a replacement type 5868T > A, occurred in the CCA-stem region in the TTGGA sequence which was a novel mutation. The second mutation, was a replacement type 5849C > T, which occurred in the V-region in the AGAC sequence as a heteroplasmic mutation. The third one, a replacement type 5836T > C, occurred in the T-stem region in the CCTCT sequence. All these regions and nucleotides were examined for conservation. We found that these regions were fully conserved, and these changes in them caused changes in the structure of tRNAs. It was also proved by examining their altered structure's Δ G that all these mutations have smaller (more stable) Δ Gs. As a result, tRNAs structure changes have affected their performance.

Every mt-tRNA has an extensive impact in various main organ functions in the body, then a mutation in their nucleotide sequence can cause several diseases. For instance, cysteine and tyrosine mt-tRNA mutations can cause musculoskeletal, central nervous system, cardiovascular, and various diseases. This has been observed in 5814A > G mutation of cysteine mt-tRNA that can lead to either encephalopathy or cardiomyopathy (26). Some diseases related to cysteine and tyrosine mt-tRNA mutations are summarized in table II (27).

Placental villous damage is probably linked to the incidence of unexplained miscarriage (28). Depending on the first 10-12 wk of conception, the plugs of extravillous trophoblast obstruct the maternal spiral arteries and prohibit maternal blood flow from penetrating the intervillous space, making a physiological hypoxia environment. Maternal arterial circulation leads to a 3-fold rise in the intervillous space, producing reactive oxygen species in the vulnerable placental villi, arising from mitochondrial systemic downregulation pathways implicated in mitochondrial redox functions and balance such as glutathione metabolism and oxidative phosphorylation. Also other research has demonstrated that mitochondrial dysfunction in the placenta, such as unbalanced placental apoptosis, has an important role in different gestation pathology, including early pregnancy loss (13). Moreover, a high level of apoptosis in decidua and placental tissue (e.g., chorionic villi) is related to abnormal embryonic development and pregnancy loss, especially IRM (22, 28, 29). In unexplained miscarriages, decreased ATP levels may be because of extreme trophoblastic cell apoptosis and extreme ATP consumption; apoptosis is an ATP-dependent procedure (28). Also, it is observed that the process of apoptosis in trophoblastic cells of the miscarriage group was significantly higher because the level of Bax was higher, while the expression level of Bcl-2 (an anti-apoptotic protein) was markedly reduced (28).

**Table 2:** Clinical phenotypes of cysteine and tyrosine mt-tRNA mutations related diseases


**Gene**	**Alternative name**	**Clinical phenotype (s)**	**PMID**
***MTTC***	mt-tRNA-Cys	MELAS; dystonia	8829635; 9185178; 17724295
***MTTY***	mt-tRNA-Tyr	Exercise intolerance; CPEO with myopathy; FSGS and dilated cardiomyopathy	11071502; 11756614; 14598342
MTTC: Mitochondrial tRNA cysteine, MTTY: Mitochondrial tRNA tyrosine, MELAS: Mitochondrial encephalomyopathy, lactic acidosis and stroke-like episodes, CPEO: Chronic progressive external ophthalmoplegia, FSGS: Focal segmental glomerulosclerosis			

## 5. Conclusion

Miscarriage is a multifactorial disease, so it is suggested that other mitochondrial genes, including all 22 mt-tRNAs, be investigated. More studies are necessary to clarify and show the primary or secondary role of tRNA mutations in embryonic development. As well, increasing statistical community and more advanced bioinformatics studies about functional structures of mt-tRNAs may be very helpful in obtaining etiological information.

##  Conflict of Interest

The authors declare that there is no conflict of interest.
